# Supplementary material for: Cloud BioLinux: pre-configured and on-demand bioinformatics computing for the genomics community
Source: BMC Bioinformatics. 2012 Mar 19;13:42. doi: 10.1186/1471-2105-13-42 (PMC3372431; doi:10.1186/1471-2105-13-42)
Supplement: Additional file 1 — Supplementary 1 Cloud BioLinux software documentation in the form of a mini, self-contained website. Users need to download and uncompress the .zip file, and open through a web browser the "index.html" file available on the main directory. (ZIP 1823 kb). [file 1471-2105-13-42-S1.ZIP › Cloud-BioLinux-Package-Documentation/Protein.html]

Bio-Linux Software Documentation Pages

Back to search form

### Protein

|  |  |
| --- | --- |
| Category | Applications |
| Protein | omssa |
| SubCategory | Applications |
| 2d\_structure | arb    einverted    garnier    helixturnhelix    hmoment    newcoils    pepcoil    pepnet    pepwheel    tmap |
| 3d\_structure | garlic    psiphi    rasmol |
| Composition | backtranambig    backtranseq    banana    btwisted    chaos    charge    checktrans    complex    compseq    compseq    dan    density    emowse    freak    freak    get-len    giep    iep    isochore    mwcontam    mwfilter    octanol    pepinfo    pepstats    pepwindowall    sirna    wordcount    wordcount |
| Dynamics | gromacs |
| Mapping | peptidemapper |
| Masking | dustmasker    segmasker    windowmasker |
| Motifs | antigenic    digest    dreg    epestfind    fuzznuc    fuzzpro    fuzztran    fuzztran    glam2    helixturnhelix    marscan    ocount    oddcomp    patmatdb    patmatmotifs    pepcoil    sigcleave |
| Mutation | msbar    msbar    pregap4    shuffleseq    shuffleseq |
| Profiles | jprofilegrid    profit    profit    prophecy    prophecy    prophet    prophet |
